# Transcriptional, proteomic and metabolic drivers of cardiac regeneration

**DOI:** 10.1136/heartjnl-2024-325442

**Published:** 2025-03-04

**Authors:** Matthew Cook, Sean Lal, Robert D Hume

**Affiliations:** 1 School of Biomedical Sciences, Faculty of Health & Medicine, University of New South Wales, Sydney, New South Wales, Australia; 2 Department of Cardiology, Royal Prince Alfred Hospital, Sydney, New South Wales, Australia; 3 School of Medical Sciences, The University of Sydney Faculty of Medicine and Health, Sydney, New South Wales, Australia; 4 Centre for Heart Failure and Diseases of the Aorta, The Baird Institute, Camperdown, New South Wales, Australia

**Keywords:** Myocardial Infarction, Cardiovascular Diseases, Heart Failure, Systolic, Heart Failure

## Abstract

Following injury, many organs are capable of rapid regeneration of necrotic tissue to regain normal function. In contrast, the damaged heart typically replaces tissue with a collagen-rich scar, due to the limited regenerative capacity of its functional contractile cardiomyocytes (CMs). However, this regenerative capacity varies dramatically during development and between species. Furthermore, studies have shown that cardiac regeneration can be enhanced to return contractile function to the damaged heart following myocardial infarction (MI). In this review, we outline the proliferative capacity of CMs *in utero*, postnatally and in adulthood. We also describe the regenerative capacity of the heart following MI injury. Finally, we focus on the various therapeutic strategies that aim to augment cardiac regeneration in preclinical animal models. These include altering transcripts, microRNAs, extracellular matrix proteins and inducing metabolic rewiring. Together, these therapies aim to return function to the damaged heart and potentially improve the lives of the millions of heart failure patients currently suffering worldwide.

## Introduction

 Cardiovascular disease (CVD) remains the leading cause of death globally, despite ongoing research into pathogenic mechanisms and novel therapies.^1^ This loss of human life also comes with a significant economic burden, with annual costs exceeding $A2.68 billion and representing 1.5% of hospitalisations in Australia.^2 3^ Within the plethora of CVDs, heart failure (HF), as a consequence of ischaemic heart disease (IHD), remains the biggest killer. Following myocardial infarction (MI), hundreds of millions of the heart’s contractile muscle cells, known as cardiomyocytes (CMs), undergo cell death via coagulative necrosis.^4 5^ Simultaneously, myocardial remodelling occurs, with inflammatory and fibrotic processes that replace dead tissue with a collagen-rich scar, reducing the heart's contractility.^5^

Historically, it was thought that adult CMs were terminally differentiated cells, without the ability to undergo proliferation or regeneration following injury. However, seminal human studies have shown low levels of CM cellular division in adults during homeostasis.^6 7^ Animal models have also challenged this dogma, showing the heart has a limited but detectable capacity for cardiac regeneration in response to damage.^8^,^10^ Many studies have attempted to elucidate and augment the underlying drivers of cardiac regeneration, with a plethora of genes, proteins, metabolites and other biochemical cues being identified. This review will discuss the proliferative capacity of CMs in utero, postnatally, in adulthood and in response to damage, focusing on emerging transcriptional, proteomic and metabolic drivers of CM regeneration post-MI. Here, we will also describe the current literature on potential HF therapies that attempt to augment this complex process.^11^

### Cardiomyocyte proliferation in development

During mitosis, CMs can undergo karyokinesis (division of cell nuclei) without completing cytokinesis (separation of cytoplasm to form two daughter cells), resulting in multinucleated CMs. Both small and large animal models show that prenatal and early postnatal hearts have a higher proportion of mononucleated CMs compared with adult hearts. As CMs undergo rapid proliferation in utero and in the early postnatal period,^12^,^15^ mononucleation of CMs correlates to an increased proliferative capacity. We have covered this subject in more detail in online supplemental material and summarised in table 1.

**Table 1:** Non-human cardiomyocyte proliferation

Species	Developmental timepoint	Mononucleated CMs	CM turnover	Regenerative capacity
Mouse^9 10 12^	Pd3	80.1%		Regeneration 21dpi
	Pd4		1% daily	
	Pd7	14.7%		Scarring 21dpi
	Pd70		0.015% daily	
Rat^13 75^	Pd1	97.1%		Regeneration 21dpi
	Pd4	82.8%		
	Pd8	41.7%		
	Pd12	9.1%		
	Pd56			Scarring 21dpi
Sheep^15 17^	Gd0-Gd90	100%		Regeneration 30dpi
	Gd145	<50%		
	Pd0	30%		
	Adult			Scarring 30dpi
Pig^14 18^	Pd1	90%		Regeneration 30dpi
	Pd2			Partial regeneration 30dpi
	Pd3			Scarring 30dpi
	Pd7			
	Pd15	50–75%		
	Pd30	10%		
	Pd60			

CM, cardiomyocyte; Dpi, days post-injury; Gd, gestational day; Pd, postnatal day.

### Cardiomyocyte proliferation in the postnatal and adult heart

**Figure 1:**
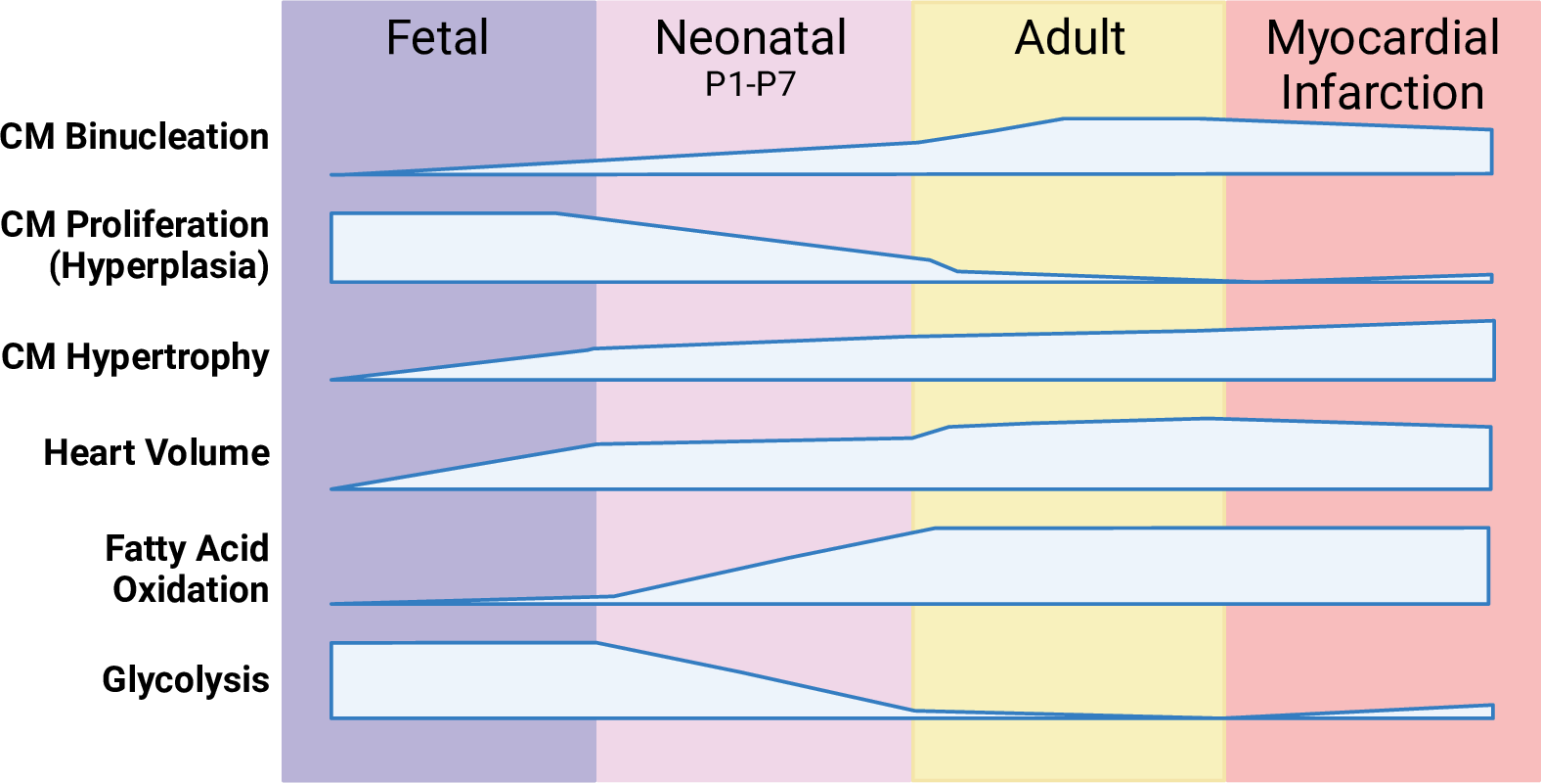
Timeline of cardiomyocyte (CM) processes during development and following adult myocardial infarction (MI) injury.

There are an estimated 2–3 billion CMs in the healthy adult human heart. Following the more gradual reduction of CM proliferation during gestation,^15 16^ CMs undergo a further notable reduction in proliferative capacity on postnatal day 7 (P7) in mice and P3 in pigs, correlated with a considerable reduction in mononucleated CMs.^9 14 17 18^ This physiological development coincides with a change from hyperplasia to hypertrophy, allowing for an increase in cardiac volume without an increase in CM numbers. This transition underscores a critical developmental shift, wherein the heart’s regenerative potential diminishes postnatally, marking a departure from the robust proliferative capabilities observed in utero (figure 1).

#### Human cardiomyocyte proliferation

Although there is a considerable paucity of literature relating to postnatal-adult human CM proliferation, several landmark studies exist. This is due to the difficulty in obtaining healthy human hearts for research, the absence of transgenic models and ethical roadblocks preventing cell labelling or lineage tracing. The scarcity of healthy heart samples is partially due to recent advances in technology and logistics, which have allowed the majority of healthy hearts to be transplanted. In other cases, they can only be obtained postmortem and thus subjected to considerable tissue degradation.^19^ Data from key human CM proliferation studies are summarised in table 2 and described in further detail within online supplemental file .^6 7 20^ Interestingly, unlike animal models, these studies have not shown a correlation between CM nucleation and age, highlighting the key differences between humans and current animal models.

**Table 2:** Human cardiomyocyte proliferation

Study	Proliferation metric	Age					
		1 month	1 year	10–20 years	25 years	40 years	70–75 years
Mollova *et al*^6^	M-Phase		0.012–0.04%	0.005–0.009%		Undetectable: 0.001%	
	Mononucleation		67.8%	63.5%			
Bergmann *et al*^20^	Annual turnover				1%		0.45%
Bergmann *et al*^7^	Annual turnover			0.8%			0.3%
	Mononucleation	~85%		~65–75%		~70%	~75%

Methods: Mollova *et al*^6^ used pHH3, a-actinin, MKLP1 analyses by confocal microscopy and laser-scanning cytometry; Bergmann *et al*^20^ used 14C cell dating; Bergmann *et al*^7^ used 14C cell dating and computerised cell counting.

### Cardiac regeneration following injury

#### Non-mammalian cardiac regeneration

Cardiac regeneration is the process in which the injured heart is repaired via the growth of new functional myocardium—the contractile portion of the heart containing viable CMs. Cardiac regeneration was first discovered in non-mammalian hearts, including newts and zebrafish.^8 21^ These models were shown to undergo myocardial regeneration through increased CM proliferation in response to both surgical resection and cryoinjury.^22^ The cellular processes occurring within these regenerative injury models initiated with erythrocyte clotting followed by fibrin formation, cardiac myofibre penetration and complete replacement of the fibrin clot with functional CMs by 60 days postresection. Moreover, CM mitosis was observed in these models, with epicardial CMs identified as the leading edge of proliferation.^8^ One pathway underlying this process was the Wnt/β-catenin signalling. This pathway is involved in scar and fibrin resolution and the dedifferentiation, proliferation and redifferentiation (DPR) of CMs in cryoinjured zebrafish hearts.^22^,^24^ Dedifferentiation is the process by which fully differentiated cells undergo structural and transcriptional changes that return the cell to a precursor-like state. In the case of adult CMs, this transition to a less mature state allows them to undergo mitosis. These injury models also found that by inhibiting endocardial Notch, an upstream inhibitor of Wnt, a reduction in CM proliferation occurred, further confirming the involvement of Wnt signalling in this process.^23^ This is one of many signalling pathways implicated in myocardial regeneration, which have been further investigated in mammals to understand their applications and potential to improve cardiac function in injured human hearts.

#### Mammalian (non-human) cardiac regeneration

Porrello *et al* were the first to show cardiac regeneration in a rodent injury model.^9^ In this landmark study, the authors undertook apical heart resections in P1 mice, surgically removing the tip of the left ventricle. Using mitotic markers phosphorylated-histone H3 (pHH3) and aurora kinase B (Aurkb), newly formed DNA marker bromodeoxyuridine (BrdU) and CM-marker cardiac troponin T (cTnT), they demonstrated the mice had replaced the resected tissue with new functional CMs and thus undergone cardiac regeneration. BrdU levels were increased adjacent to the site of injury, known as the border zone (BZ), by a factor of 7, thereby showing the regenerative process was enhanced within CMs adjacent to the damaged myocardium. Immunohistochemical analysis at day 7 postinjury showed new CMs had disorganised sarcomeric structures, suggesting a disassembly indicative of dedifferentiation and mitosis. At 2 months postresection, echocardiography confirmed that these hearts had preserved contractility, demonstrating the new tissue that regenerated was functional. This study also showed that when the same procedure was undertaken on 7-day-old mice, their hearts failed to regenerate CMs and instead developed significant fibrosis. This highlights that the regenerative potential of the rodent heart following apical resection is limited to a brief neonatal window. This may be related to the physiological growth response during this period, before the proliferative switch from hyperplasia to hypertrophy occurs.

**Figure 2:**
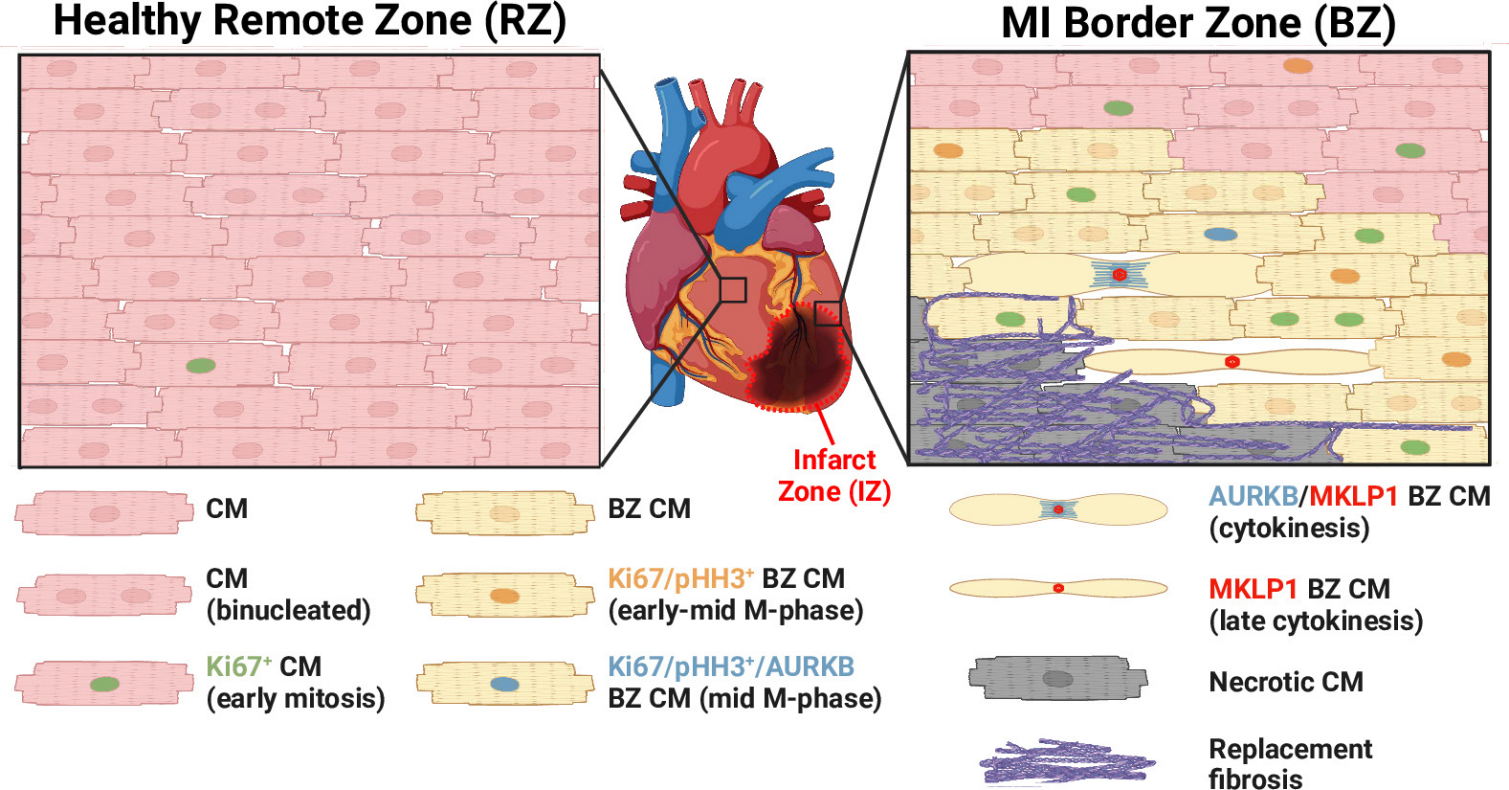
Schematic of post-myocardial infarction (MI) cardiomyocyte (CM) proliferation in the infarct remote zone (RZ) and border zone (BZ). CMs frequently undergo incomplete mitosis in the uninjured heart, resulting in binucleation. Following MI, BZ CMs undergo increased mitosis with increased completed cytokinesis, resulting in increased CM proliferation within the BZ (albeit at low levels with no detectable increase in cardiac function).

Multiple murine studies have proposed that DPR is required for CM cell cycle re-entry post-MI. Zhang *et al* used gene cell-fate mapping, CM lineage tracing and dedifferentiation reporting to exclude the possibility of a non-CM progenitor pool.^25^ Stem cells ordinarily differentiate and become increasingly more lineage restricted until they become terminally differentiated. However, during DPR adult CMs dedifferentiate into a precursor-like state. Zhang *et al* found a significant increase in dedifferentiated CMs post-MI, which displayed a smaller, rounder and non-functional phenotype compared with typical adult CMs. These dedifferentiated CMs exhibited higher rates of cell cycling, indicated by increased Ki67 expression. These rodent findings indicate the importance of DPR for increased CM proliferation following MI (figure 2).

#### Human cardiac regeneration models

There is minimal evidence of myocardial regeneration in humans. One case study reported a newborn sustaining a severe MI and subsequently undergoing full functional recovery over a period of 2 weeks.^26^ Importantly, our new unpublished evidence^27^ (preprint) also suggests adult humans increase cardiomyocyte proliferation post-MI. As patients who suffer MI are of an average age of 59 years,^28^ evidence of their regenerative potential is pivotal for the development of therapies that aim to augment cardiac regeneration, which would have long-lasting benefits for the remainder of these patients lives.

### Transcriptional and proteomic drivers of post-MI cardiac regeneration

Developing an understanding into the genetic drivers of cardiac regeneration is essential in appreciating how these pathways result in physiological and functional responses to MI. These networks are integral in aiding the development of novel HF therapeutics that target pro-regenerative pathways.

#### Hippo/yes-associated protein signalling

Yes-associated protein (YAP) is a transcriptional cofactor that exerts pro-proliferative and antiapoptotic effects on CMs by activating insulin-like growth factor (IGF) and Wnt signalling.^29 30^ However, YAP activation of these pathways is ordinarily blocked in adult CMs by the Hippo pathway. Post-MI mouse studies have shown that *Yap* overexpression, by adenoviral-associated vectors (AAV) or Hippo blockade, results in increased CM proliferation and survival post-injury, while *Yap* KO causes worsened regeneration, ventricular wall thinning, dilated cardiomyopathy (DCM) and fibrotic infarct zones.^29 30^ In these studies, *Yap* overexpression also resulted in increased CM size with some disorganised structures, while *Hippo* blockade resulted in cell elongation with no decrease in maturation markers. This highlights CM regeneration may be possible without DPR. These findings were corroborated by a study using catheter-mediated subendocardial injection (CMSI) of AAVs that induced *Hippo* knockdown in pigs, post-MI.^31^ In this study, the *Hippo* pathway gene *Salvador* (*Sav*) was inhibited in the BZ of CMs by RNA-incorporated AAVs, delivered through mesoporous silica nanoparticles (MSNs), thereby allowing for Yap-induced regeneration. This increased the proportion of Aurkb positive and mononucleated CMs and a 14.3% improvement in ejection fraction at 3 months post-MI. Dedifferentiation was also noted in CMs with positive proliferative markers, supporting the DPR model. This large animal model is important in the translation of both *Hippo* blockade and CMSI to human studies as a safe and effective means of stimulating CM regeneration.

#### Hedgehog signalling

Hedgehog (HH) is a paracrine signalling pathway downregulated by P7 and coordinates cardiac progenitor proliferation.^32^ The HH ligand, sonic HH (Shh), is expressed in endothelial and smooth muscle cells, with paracrine signalling on neighbouring CMs via the smoothed membrane receptor (Smo).^32^ In post-MI *Smo* KO mice, impaired CM proliferation, increased scar size and decreased cardiac function are observed. Conversely, increased HH signalling reduces infarct fibrosis and increases CM cell cycling and proliferation. Thus, HH activation is sufficient to induce cardiac regeneration.

#### IGF 2 binding protein 3

IGF 2 binding protein 3 (IGF2BP3) signalling has important roles in regulating CM proliferation and cardiac regeneration in mice, with decreasing postnatal expression levels coinciding with decreasing CM proliferative capacity.^33 34^ IGF2BP3 achieves its effects via post-transcriptional gene regulation, specifically by binding to multiple target mRNAs. Although originally identified as binding to and altering IGF2 mRNA translation,^35^ its proregenerative effects have been identified via its binding and stabilising of matrix metalloproteinase 3 (MMP3) mRNA and subsequent downstream effects on CM proliferation.^34^ Similar to IGF2BP3, MMP3 also shows reduced postnatal protein expression during postnatal development. Conversely, both proteins show increased post-MI expression in both mouse models^34^ and within the largest comprehensive human post-MI single-cell spatial-omic study to date.^36^ This suggests that while IGF2BP3 activity is dormant in adult CMs, it is an MI-responsive gene. Furthermore, overexpression of *Igf2bp3* in MI murine models demonstrates pro-regenerative effects via increased CM proliferation, reduced infarct size, improved heart function and improved survival.^34^

#### Troponin I interacting kinase

CMs can increase their DNA content to carry more than two sets of chromosomes in one cell and are termed polyploid. CM ploidy varies between species, ages and populations and has been implicated in their ability to undergo regeneration.^37^ A study of adult mice hearts found that those with a higher proportion of CMs with two sets of DNA within a singular CM nucleus (mononuclear diploid CMs (MNDCMs)) were associated with improved post-MI outcomes, over those with polyploid CMs.^37^ The CM-specific gene, *Tnni3k,* was identified as having a strong inverse association with MNDCMs, which is elevated in P1-7 mice, correlating with the transition from mononucleated CMs to binucleated and multinucleated CMs. *Tnni3k* KO mice exhibit increased MNDCMs and post-MI CM regeneration, while *Tnni3k* overexpression increases CM ploidy and decreases regenerative capacity. It is thus important to consider species and demographic variations in CM ploidy and the implications on their regenerative potential.

#### MicroRNAs

MicroRNAs are non-coding RNAs that regulate gene expression by binding mRNAs to subsequently degrade (more frequent) or stabilise (less frequent) them, resulting in either mRNA suppression or activation, respectively.^38^ The last two decades have seen an emergence of studies investigating the role microRNAs have on both MI and cardiac regeneration.^39 40^ For example, an early rodent study showed administration of microRNA-199a via AAV vectors induced adult CM proliferation and near-complete cardiac regeneration post-MI with complete functional recovery.^41^ However, when translated into a pig model, although functional and physiological improvements were seen early, sudden arrhythmic deaths occurred 7 weeks after treatment.^42^ This was attributed to excessive microRNA expression and uncontrolled cell proliferation, raising concerns for the AAV method of therapeutic delivery. To address this, a recent study used microRNA-199a-coated nanoparticles to deliver a single dose into infarcted rat myocardium.^43^ This therapy was both preventative against MI, protective against post-MI injury and likely safer than continuous microRNA expression via AAV transduction. However, although this nanoparticle study showed important improvements in cardiac function, it did not quantify CM proliferation or cardiac regeneration. Thus, functional improvement via mechanisms other than cardiac regeneration may have occurred.

While some microRNAs can increase CM proliferation and cardiac regeneration, others have shown the converse effect. For instance, the microRNA-15 family,^44^ microRNA-128^45^ and microRNA-25^46^ all attenuate CM proliferation and cardiac regeneration. As such, inhibition or loss of these microRNAs can have pro-regenerative effects and may be an alternative therapeutic target.^44 45^ Although microRNAs hold promise as a cardiac regeneration therapy, studies with longer timepoints and improved delivery methods are required to confirm their safety and efficacy before translation is possible.

#### Extracellular matrix proteins

**Figure 3:**
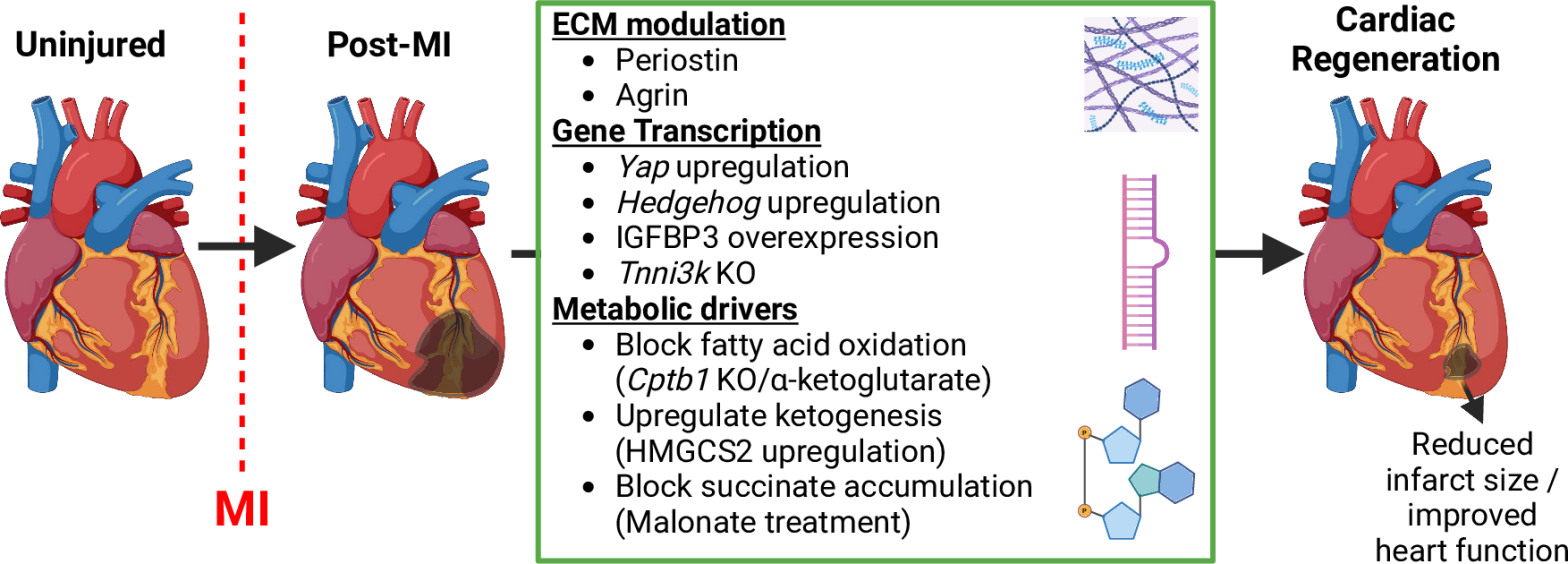
Post-myocardial infarction (MI) cardiac regeneration therapies. Extracellular matrix (ECM) proteins, transcripts and metabolic drivers tested in preclinical animal models that induce post-MI cardiac regeneration.

The cardiac extracellular matrix (ECM) composition undergoes considerable changes throughout in utero development, when CMs are most proliferative, to postnatal maturation, when CMs exhibit limited proliferative capacity.^13 47 48^ Furthermore, the cardiac ECM undergoes distinct changes post-MI, with inflammation, proteolysis and fibrotic scar formation significantly altering infarct and BZ ECM expression.^49 50^ An early study identified the ECM protein periostin as an inducer of proliferation and CM repair.^51^ Periostin is a non-structural matrix protein that shows increased expression in the embryonic^52^ and post-MI heart.^53^ Administration of periostin into healthy rat myocardium was shown to induce complete mitosis of adult CMs, acting through integrin receptor and phosphatidylinositol-3-OH-kinase signalling pathways.^54 55^ Evidence of its regenerative properties was demonstrated when post-MI administration of periostin resulted in improved cardiac function,^51^ while others showed genetic KO of periostin in mice inhibited post-MI regeneration.^56^ Another ECM protein that has been linked to cardiac regeneration is agrin; a large heparan sulphate proteoglycan.^57^ Agrin is expressed at high levels within the myocardium P1 and declines rapidly from P1 to P7, correlating with the decline in CM proliferative capacity. In an MI injury model, administration of agrin into infarcted mouse hearts not only increased CM proliferation but also improved heart function, a tenet of cardiac regeneration. This further supports the therapeutic application of ECM proteins to augment CM proliferation in an injury setting (figure 3).

### Metabolic drivers of cardiac regeneration

#### Carnitine palmitotransferase (Cptb) and α-ketoglutarate

In utero, CMs predominantly use glucose as their main energy source.^58^ Following postnatal CM maturation, there is a switch from glycolysis towards fatty acid oxidation, which coincides with the reduction in CM proliferative capacity. A recent study has shown that a major driver of this metabolic switch is the muscle-specific isoform of carnitine palmitotransferase (*Cpt1b*).^58^ Interestingly, *Cpt1b* KO mice subjected to MI show virtually absent scars 3 weeks post-MI, with BZ CMs identified as re-entering the cell cycle. This study also identified an associated rise in α-ketoglutarate (αKG) within CMs, a cofactor for histone demethylases and metabolite of the citric acid (tricarboxylic acid cycle; TCA) cycle. Subsequent experiments showed this accumulation of αKG activated H3K4me3-specific demethylase, which reduced H3K4me3 genes required for CM differentiation and maturation. Ultimately, *Cpt1b*-deficient CMs were converted into a more immature, proliferation-competent state, in concordance with the process of DPR. By inducing a CM metabolic switch, the authors caused CMs to dedifferentiate, proliferate and regenerate post-MI hearts, a promising therapeutic avenue for reversing ischaemic myocardial injury. Another study supported these findings by directly investigating the regenerative effect of intraperitoneal αKG supplementation in mice.^59^ Post-MI αKG administration extended the CM regenerative window, reducing infarct size and improving ejection fraction, fractional shortening and dilatation. Both studies showed that αKG increased regeneration by upregulating proliferation-related pathways mediated by JMJD3-related demethylation of H3K4me3. Thus, metabolic rewiring can induce CM proliferation via epigenetic alteration of CM maturity to induce DPR.

#### Metabolic influence of heart rate

Increased heart rate (HR) is an independent risk factor for ischaemia, remodelling and chronic HF, which may be explained by its metabolic influence.^60^,^62^ A mouse study has shown elevating HR increases the ADP to ATP ratio in CMs, indicating a lower proportion of high-energy molecules, due to increased energy demand.^63^ Conversely, reducing HR decreased AMP/ATP ratio, indicating a higher proportion of high-energy molecules, suggesting lower energy demand. In addition, HR reduction upregulated glycolytic enzyme expression, including HK2, G6PD, PFKFB3, GAPDH and PKM2. These findings suggest decreasing HR increases CM glycolysis; the predominant form of cardiometabolism in the embryonic heart. Interestingly, this induced increased CM cell cycle re-entry and proliferation. To investigate whether this mechanism could be manipulated to produce regenerative outcomes, the researchers slowed post-MI HR using negative chronotropic therapies administered by subcutaneous osmotic pump in mice. This increased CM proliferation in BZ and remote infarct zone, increased systolic function, reduced fibrosis and reduced mortality. With chronotropic therapies already commonly in use, this stimulation of glucose metabolism and cardiac regeneration by a pharmacological reduction of HR may be easily implemented into future clinical trials.

#### 3-hydroxy-3-methylglutaryl-CoA synthase 2

The effects of altered cardiometabolism in cardiac regeneration have been investigated by focusing on the metabolic changes occurring during induced cell reprogramming.^64^ In one such study, the authors induced CM reprogramming in mice using Yamanaka factors (Oct4, Sox2, Klf4 and c-Myc). This subsequently led to CM DPR and a metabolic switch to ketogenesis. Interestingly, the rate-limiting enzyme of ketogenesis, 3-hydroxy-3-methylglutaryl-CoA synthase 2 (Hmgcs2), was shown to be highly overexpressed in these mice. By overexpressing Hmgcs2 in vivo using AAV in post-MI mice, the authors demonstrated that upregulated Hmgcs2 alone can induce CM proliferation and cardiac regeneration without the need for Yamanaka factors. This study highlights how metabolic reprogramming to predominantly ketogenesis through Hmgcs2 induction can permit DPR and cardiac regeneration following MI or hypoxia.

#### Malonate

Succinate and malonate are metabolites that are involved in aerobic respiration.^65^ Specifically, succinate is the rate-limiting step in the citric acid (TCA) cycle required for energy metabolism, as it is converted into fumarate by succinate dehydrogenase (SDH). Malonate is an inhibitor of SDH and therefore prevents aerobic respiration through the TCA cycle. To investigate the effects of increasing TCA cycling following MI, a group injected succinate into neonatal mice.^65^ This study showed succinate increased DNA damage, impaired cardiac function and reduced both CM proliferation and heart regeneration, therefore shortening the neonatal regenerative window. To investigate whether blocking the TCA cycle had the opposite effect, the investigators administered malonate to inhibit SDH and consequently extended the regenerative window in postnatal hearts. Importantly, post-MI malonate treatment in mice induced CM proliferation, increased myocardial thickness, reduced fibrosis and improved cardiac function, all hallmarks of cardiac regeneration. This demonstrates that post-MI cardiac regeneration via metabolic reprogramming is possible using malonate. As this metabolite occurs naturally in fruit such as grapes and strawberries,^66 67^ its rapid clinical translation could hold promise, likely circumventing some of the regulatory framework required for newly formulated compounds.

#### Sphingolipid metabolism

Sphingolipids are a class of lipids derived from amino and fatty acids, and their metabolic role in heart regeneration has been a recent focus. One study investigated the opposing regenerative effects of sphingolipid isoenzymes SphK1 and SphK2, which both produce the metabolite sphingosine-1-phosphate.^68^ SphK2 has higher rates of expression at birth, which declines in the early neonatal period, coinciding with a decrease in proliferative capacity, whereas SphK1 expression increases into adulthood and is associated with a lower proliferative capacity. Stimulating SphK2 expression via intramyocardial injection of engineered AAVs and pharmacological inhibition of SphK2 both resulted in regeneration of infarcted adult mice hearts, with increased mononucleated CMs and decreased binucleated CMs. Cotreatment with both methods had an additive effect in reducing fibrotic scarring and increasing myocardial tissue size, with functional benefits in ejection fraction(EF), fractional shortening and left ventricular thickness, arising in the subacute (>2 weeks) but not acute (<1 week) period. This challenges previous studies, which have required glycolytic metabolism for CM proliferation, instead highlighting that modified lipid utilisation, through enzymatic augmentation, as an alternative means of stimulating cardiac regeneration.

### Enhancing translation to human studies

Many preclinical studies attempting to increase CM proliferation have resulted in an increase in regeneration. However, it is important to emphasise the biological and clinical significance of these changes. Further validation against other pleiotropic mechanisms is crucial to evaluating translation to human studies. For instance, studies often show a discrepancy between CM proliferation and functional improvements, with minimal increases in CM numbers, leading to a disproportionate improvement in cardiac function and reduction of infarct size. Furthermore, some pro-regenerative therapeutics may alter post-MI remodelling through other unintended mechanisms. For example, we have previously shown therapeutic improvement in post-MI heart function through altered scar characteristics but no decrease in scar size.^69 70^ These desirable but ‘off-target’ effects must be considered when testing pro-regenerative therapeutics.

#### Targeted administration

During translation to large animal and human models, there is an increased emphasis on ensuring safety in administration, while quantifying cardiac and off-target adverse effects. One study undertaking AAV Hippo knockdown via CMSI localised targeting of the post-MI BZ has shown to have no increase in local inflammation despite its invasive means as well as no off-target tumours.^31^ However, while CMSI AAV administration anatomically targets the area of infarction, it is not CM-specific and does not allow tunable expression of AAVs. Another means of delivery include lipid nanoparticles (LNPs), which deliver RNA or proteins to target cells without a viral vector.^71^ A recent study delivered LNPs by intramyocardial injection, but found that epicardial cells, epithelial cells and fibroblasts were targeted over the desired CMs and smooth muscle cells. Distribution to other organs was also noted, with transfection to the liver, spleen and lungs. Therefore, the development of new CM-specific AAV capsids is essential, which may allow for a less invasive means of targeting CMs and avoiding non-CM cardiac cells.

#### Tuneable expression

There has been significant focus on the use of AAVs, which incorporate new genetic material into host DNA and allow for the examination of short-term benefits. However, the development of tuneable expression, by which the increased proliferative capacity of CMs can be moderated, is essential to prevent persistent expression, leading to hyperproliferation and potentially fatal adverse effects seen previously.^42^ A previously mentioned study by Liu *et al*^31^ used MSNs to deliver AAVs in a safe and effective manner; however, metrics of CM proliferation were not quantified. While this therapy was not tunable, MSNs are non-viral capsids and a potential means of delivering short-term therapeutic interventions without ongoing transcription, such as messenger RNA. The emergence of targeted, tuneable approaches to CM regeneration highlights the potential for progression to human trials.

#### Patient population

The vast majority of the aforementioned studies have investigated the use of regenerative therapies in the context of acute MI. However, this fails to answer the need for IHD and chronic HF therapies. The effectiveness of these treatments in the delayed setting should also be investigated, to assess their application to patients with HF, and the effectiveness of novel therapies should be stratified based on acute or chronic IHD.

### Conclusion and the future of cardiac regeneration therapies

Following decades of unsuccessful research trying to identify and manipulate a resident cardiac stem-like cell source capable of regenerating the heart, interest in cardiac regeneration had waned considerably.^72^ However, with focus shifting towards reactivating adult CM proliferation or induced pluripotent stem cell (iPSC)-derived CM therapies, the field has seen a resurgence over the last decade.^9 57 58 65^ These discoveries have opened exciting possibilities for treatments aimed at amplifying the heart’s regenerative capacity. Despite this potential, all effective therapies are currently preclinical, with a minority progressing to large animal models and none yet in clinical trials. Moreover, in some cases, considerable adverse effects have been identified that need to be resolved for the future safety and application of these novel therapies.^42^ We must also improve translation to humans, potentially with long-term preclinical studies and novel human *in vivo*^73^ and *in vitro*^74^ models. With current preclinical treatments covering a wide range of biological approaches (ie, ECM modulation, microRNAs, AAV gene delivery, metabolic reprogramming) ensuring effective yet safe delivery methods is paramount. Overall, the future of cardiac regeneration is unknown but promising, with considerable potential to improve and save lives.

## Supplementary material

10.1136/heartjnl-2024-325442 online supplemental file 1
