# Supplementary material for: Transcriptional, proteomic and metabolic drivers of cardiac regeneration
Source: Heart. 2025 Mar 4;111(18):e325442. doi: 10.1136/heartjnl-2024-325442 (PMC12418534; doi:10.1136/heartjnl-2024-325442)
Supplement: online supplemental file 1 [file heartjnl-111-18-s001.docx]

Online Supplement

**Cardiomyocyte proliferation *in utero***

The heart's developmental journey begins during embryogenesis. A rapid increase in embryo size requires early formation of the heart, making it the first functional organ in the growing fetus^1^. Cardiac cells arise from the precardiac mesoderm and during gastrulation these cells migrate to form bilateral paired heart fields^2-5^. By gestational day 10, a primitive structure known as the heart tube is formed and has pulsatile activity from around day 22. The embryonic heart then achieves its distinctive four-chambered structure by the end of gestational week 7 in humans, embryonic day 12.5 (E12.5) in mice, E17.5 in rats, E32.5 in pigs and E33 in sheep^4-6^. This developmental timeline is relatively conserved between mice and humans (when accounting for obvious differences in gestation time, i.e. 20 vs 280 days), exhibiting structural parallels from murine embryonic day 9.5 to birth corresponding to human Carnegie stages 13-23^5, 7^. This makes mice models advantageous for investigating the processes of cardiac proliferation and regeneration, as the cellular signalling occurring in murine heart formation, known as cardiogenesis, parallels many processes occurring in humans. Other mammalian models including sheep and pigs are also used, albeit less frequently, as they share other developmental and physiological processes similar to human cardiogenesis. However, large animal models also include longer gestational times, increased costs and limited transgenic models, reducing their frequency in embryonic studies.

Throughout embryonic development, a pool of cardiac progenitor cells (CPCs) undergo differentiation into the resident cell types of the heart, including contractile CMs and non-CM populations, such as endothelial cells, pericytes, fibroblasts, neurons and resident immune cells^8, 9^. This results in hyperplastic growth, contributing to the structural maturation of the heart *in utero*^10^. Most notable among these cell types and essential for cardiac function are the numerous new CMs that are formed. These contractile cells ensure the heart's ability to pump and transport blood, oxygen and nutrients throughout the body. Together, these cells work in concert during cardiac development (cardiogenesis), a process that is tightly regulated by a cascade of cellular signalling pathways and results in significant CM differentiation and proliferation^9^.

During development, CPCs undergo differentiation into CMs, followed by their maturation and reduced capacity to proliferate^9, 11, 12^. Thus, understanding CM differentiation and maturation is integral for the understanding of CM proliferation and regenerative capacity. For mesodermal differentiation and the early stages of gastrulation, mesoderm posterior homolog 1 (*Mesp1*) is the earliest sign of cardiovascular development^13-15^. Homozygous disruption of this gene has been shown to delay cellular migration of CPCs to the heart fields. While this causes structural abnormalities, the myocardium and endocardium maintain adequate differentiation, indicating that this cellular signal is not essential for CM formation or proliferation^13, 14^. Other downstream signalling pathways involved in cardiogenesis include *Nkx2.5* in the formation of the first heart field (FHF), and *Nkx2.5*, *Gata4* and *Isl1* in the second heart field (SHF)^9^. This highlights that cardiac developmental genes drive both CM differentiation and structural cardiogenesis.

One approach to determine rates of *in utero* CM proliferation is immunohistochemical (IHC) analysis of embryonic hearts at multiple developmental timepoints. IHC allows the identification of CMs and their expression of cell cycle markers, such as Ki-67 (G1 to M-phase), phospho-histone H3 (pHH3, early/mid M-phase) and aurora kinase B (AURKB, mid/late M-phase). While these cell cycle markers are provide a quantifiable metric of CM cell cycling, they require accompanying evidence of bona fide cell division. These included multi-isotope imaging mass spectrometry (MIMS), which uses stable isotopes and high-resolution imaging to confirm cell division, as well as lineage tracing and CM cell counting. Clinically-significant cardiac regeneration may also be measured using echocardiography to calculate ejection fraction (EF), ventricular wall thickness and dilatation.

IHC allows the identification of bi- or multi-nucleated CMs that have undergone karyokinesis (nuclei division) but failed cytokinesis (cytoplasmic/cell division). One study of sheep hearts showed from mid (day 90) to end (day 145) gestation, the ratio of mononucleated left ventricular CMs decreased from 100%, to less than 50%^16^. This aligns with other large animal studies^17, 18^, which have shown that *in utero* CMs undergo a significant reduction in their capacity to successfully complete mitosis via cytokinesis to form mononucleated daughter cells. Interestingly, the rates of binucleation over this same period showed an earlier peak for the left ventricular cell population in these studies, compared to the right ventricle, which indicates there are proliferative nuances between heart chambers for *in utero* CMs. Rodent models indicate that 100% of CMs are mononucleated at P1, which likely reflects their state *in utero*^10, 19^. Although CM nucleation varies between species, all species show a reduction in mononucleated CMs during development that correlates with a reduction in their proliferative capacity.

Other important signalling pathways identified in the process of CM differentiation during foetal cardiogenesis include the Notch pathway, which inhibits CPC proliferation in foetal cardiogenesis^20^. This inhibition allows CMs to undertake their functional role as a contractile tissue and has a pivotal role in SHF-derived structures. Inactivation of Notch leads to arrested looping heart morphogenesis and hypoplasia of the right ventricle, with an increase in progenitor proliferation. These anti-proliferative effects are mediated by downstream inhibition of the Wingless-related integration site (Wnt)/beta (β)-catenin proliferation pathway^21-24^. Indeed, upregulation of Wnt signalling in mature CMs can induce cell cycle re-entry^25^. Knockout (KO) of *Axin2*, a negative regulator of Wnt, enhances Wnt and bone morphogenic protein 4 (Bmp4) signalling, whilst rescuing cardiac differentiation arrest^21^. Through Wnt/β-catenin and Bmp4 signalling, they activate specific, non-overlapping cardiac-specific genes in cardiac progenitors. These include *Nkx2.5*, *Isl1*, and *Baf60c*, controlled by Wnt/β-catenin, while *Gata4*, *SRF*, and *Mef2c* are controlled by Bmp signalling. Bmp proteins have also been shown to promote proliferation by preventing CM differentiation^26^. For example, myocardial *Bmp2* overexpression was found to increase the expression of CM pro-proliferative genes, such as *Hey2*, *n-Myc*, *Id2* and *Tbx20*. Together, this cascade of *Wnt* modulators influencing CM proliferation and differentiation offer multiple therapeutic targets for cardiac regeneration,, however, its multifaceted role post-MI make its application complex^27^.

The use of embryonic stem cell-derived (ESC) or induced pluripotent stem cell-derived (iPSC) cardiomyocytes as an *in vitro* human model of cardiogenesis has gained popularity over the past decade^28-31^. For example, *NKX2.5* KO ESC cells ^32^ exhibit impaired CM formation and abnormal contractile patterns during CM differentiation. This impaired differentiation can be rescued by *HEY2* expression, demonstrating *NKX2.*5 as a critical component of CM maturation and *HEY2* as a key downstream transcriptional mediator for its function. Overall, embryonic studies demonstrate the complex signalling pathways that influence CM differentiation and proliferation *in utero* and the reduction of CM mitosis during gestation.

**Cardiomyocyte Proliferation in the Postnatal and Adult Heart**

Similar to *in utero* development, the proportion of postnatal CMs undergoing karyokinesis without cytokinesis further increases from end gestation, resulting in an increased formation of bi- or multi-nucleated polyploid CM nuclei^53^. Bromodeoxyuridine (BrdU) labelling of newly synthesised DNA in postnatal rats demonstrates this, showing a rapid switch from proliferation to binucleation between P3 and P4^27^. This coincides with the switch from hyperplasia to hypertrophy and is considered to be an adaptive response to increased mechanical load in neonatal life^54, 55^. By identifying CM nuclear characteristics and mitotic state across samples from differing age groups, the baseline proliferative ability of CMs in the heart may be estimated and the transition to postnatal hypertrophy may be further understood. Appreciating these dynamics in cardiac development is crucial for developing strategies to harness regenerative capacities for therapeutic interventions in later stages of life.

Postnatal changes associated with reduced CM proliferation include the downregulation of cell cycle progression markers cyclin-dependent kinases (CDK), MYC and E2F, and upregulation of cell cycle inhibitors p21 and p27^10, 33^. This reduced proliferative rate also coincides with a metabolic switch from predominantly glycolysis in the foetal heart to predominantly fatty acid oxidation that occurs shortly after birth in mice and rats^34^, however the exact timing in humans is unknown^35-37^. Energy demand, oxygen concentration and mitochondrial fatty acid uptake are known influences on energy metabolism within the heart, which changes drastically from *in utero* to *ex utero*^36, 38^. A study using electron microscopy has revealed that binuclear CMs have more glycogen granule stores than mononuclear CMs, suggesting that mononuclear CMs more readily undergo glycogenolysis for glycolytic metabolism^39^. This coincides with reduced utilisation of metabolic glycolysis in binuclear CMs. This role of energy metabolism in the CM cell cycle has been an area of investigation in recent years, as modulation of these mechanisms may give insight into the influence of energy metabolism on cardiac regeneration^40-42^ (see ‘Metabolic Drivers of Cardiac Regeneration’ section).

The estimated baseline proliferative rate of postnatal and adult CMs varies between studies, due to differing animal models and means of identifying cell turnover. One seminal study by Senyo et al. used the non-radioactive stable isotope tracer, nitrogen-15-thymidine (^15^N-thymidine) to label newly formed DNA and trace cycling CMs in mice during postnatal development. These cells were then imaged using multi-isotope imaging mass spectrometry (MIMS); a complex technique that permits subcellular resolution imaging to identify proliferative CMs, as a direct means of quantifying CM turnover^43^. Using this approach, they showed that daily CM turnover reduced from 1% in newborns to 0.015% in young adult mice and 0.007% in old adult mice, demonstrating a greater than 66-fold reduction in CM proliferative capacity during postnatal development. Additionally, the mice used in this study were genetically modified to irreversibly express green fluorescent protein (GFP) in CMs when exposed to tamoxifen, allowing for pulse-labelling of cells. By using GFP labelling, the source of these new CMs was confirmed to be pre-existing CMs in the heart, rather than from a CM progenitor or adult stem cell pool, as the amount of GFP expressed in ^15^N^+ve^ CMs was virtually identical to that of surrounding ^15^N^-ve^ CMs. These results contrast with previous controversial studies, many that have been retracted, that claimed the existence of resident CPCs in the adult heart as a source of new CMs^44, 45^.

**Human Cardiomyocyte Proliferation**

One seminal human study utilised IHC to identify mitotic CMs in heart tissue sections from healthy individuals of different ages. Using this approach, Mollova et al. showed 0.012% of all CMs were in the M-phase (mitosis) in the first year of life^46^. This significantly reduced to 0.001% M-phase CMs by 40 years of age; a 12-fold decrease. Although this gave an indication of proliferative capacity, CMs may undergo karyokinesis without cytokinesis^47^. Thus, the authors also utilised cytokinesis marker mitotic kinesin-like protein-1 (MKLP-1) to identify *bona fide* cell division. This showed cytokinesis reduced from 0.015% in the first year of life, to 0.005% at 10-20 years old and to an undetectable level above 20 years old. Mollova et al. also predicted yearly CM turnover whilst controlling for multinucleation and polyploidisation, which decreased from 100% in the first year of life to 4.5% by ages 1-10 and 0.04% for 40 years and above^46^. Notably, although the percentage of mononucleated CMs did not vary significantly in all age groups, the ratio of polyploidal, mononucleated CMs increased with age. Furthermore, CMs in M-phase were predominantly mononucleated, which is consistent with findings in a previous mouse model^48^. However, another mouse model identified a transition from mononuclear to predominantly binuclear CM with age^49^. Although mouse studies are the backbone of *in vivo* studies, this incongruence between humans and mice indicates caution when making biological inferences between the two species.

Two seminal studies by Bergmann et al. also quantified adult CM turnover in adults whilst utilising an innovative and creative approach^50, 51^. These studies utilised human hearts labelled with carbon-14 (^14^C) due to environmental contamination during 20^th^ century nuclear bomb testing. The age of each CM within 12 hearts was determined by its nuclear ^14^C content and was compared to the standard curve of atmospheric ^14^C by year. The first study estimated the annual proliferative rate of the adult human CM to be 1% for 25-year-olds and 0.45% for 75-year-olds^50^. Using mathematical models for the kinetics of DNA synthesis, they also estimated that 60% of CMs present at age 60 were present at birth. The second study used both ^14^C dating and computerised cell counting under a microscope (stereology) to estimate CM turnover in 51 subjects aged 8-75^51^. They estimated that CM turnover in the left ventricle decreased from 0.8% at 20 years to 0.3% by the age of 75. The differences between these studies may be due to small sample sizes and alternative analysis techniques. Interestingly, they noted that the CM cell death is inversely proportional to age, and the number of CMs remains constant through the human lifespan despite fluctuations in endothelial and mesenchymal cell counts. While there was a higher density of CM nuclei per square millimetre in subjects under the age of 10, the nuclei per CM count was not significantly different between young and old hearts. These results were consistent with previous estimates by Mollova et al.^46^ This further highlights the process of hypertrophy in heart maturation. Additionally, while polyploidisation has not been proven to be a function of human heart aging, there is a notable decrease in CM proliferation with increased age, indicating that the overall turnover of CMs decreases with age. When comparing to the aforementioned Mollova et al study^46^, the trend of decreasing proliferative capacity is congruent, however Bergmann, et al.^51^ identified a higher estimated proliferative rate in both the younger and older populations. This highlights a difference in the methods of estimation, as Mollova et al. looked at active mitosis observable with mitotic markers, whereas Bergmann, et al. assessed the retrospective ‘birth date’ of CMs using carbon dating. Regardless of their differences, nearly a decade on, these studies remain the most comprehensive analyses of adult human CM proliferation in healthy hearts to date.

Various mouse models and experimental tools are available to elucidate CM cell fate, allowing tracing of proliferating CMs and their progeny. Until recently, a comparable human model was not available. To develop such a model, Yester et al.^52^ used a similar strategy to the aforementioned Senyo et al. study^43^, by administering the non-toxic stable isotope N^15^-thymidine to permanently label proliferating cells and their progeny. Specifically, N^15^-thymidine was administered to pregnant women carrying foetuses suffering from the congenital heart disease tetralogy of Fallot (ToF). As ToF requires postnatal corrective heart surgery to remove a portion of myocardium, the authors were able to postnatally biopsy *in utero*-labelled cardiac tissue and perform MIMS imaging to identify proliferative CMs. Although this patient population has higher rates of CM multinucleation and therefore may not reflect CM proliferation in the general population, this revolutionary technique opens new possibilities when considering human CM proliferation studies and tracing their progeny.

**Cardiac Regeneration Following Injury**

*Mammalian (Non-Human) Cardiac Regeneration*

Whilst early apical resection models^11^ were essential in furthering understanding into neonatal cardiac regeneration, it was yet to be elucidated whether similar processes occur post-MI in adult hearts. To determine this Senyo, et al. used proliferative cell labelling and MIMS to show increased CM mitosis in an 8-week-old mouse post-MI model, demonstrating an increased regenerative potential in response to injury^43^. At 8 weeks post-MI, 3.2% of CMs adjacent to the infarction underwent division. 14% of which were mononucleated and diploid, consistent with division of pre-existent CMs and therefore regeneration. This increased mitotic activity was approximately 23 times that observed in sham-operated mice. Although the majority of CM cell cycle entry led to polyploidisation and hypertrophy, this study still demonstrated that intrinsic CM regeneration is possible in mice beyond the neonatal period. Thus, this study provides evidence that while optimal regeneration occurs in the neonatal period, post-MI CM proliferation can occur in the adult heart (Figure 2). Importantly, although detectable, the proportion of CM proliferation was not sufficient to provide measurable improvements in heart function.

Intercellular communication between CMs has been implicated in DPR. For example, Wang et al. showed that 7% of adult mouse CMs actively dedifferentiated and proliferated when co-cultured with neonatal rat CMs^53^, suggesting immature CMs release signalling molecules that induce DPR. In this study, mononucleated CMs were more likely to proliferate than multi-nucleated CMs and the resulting cytokinetic division of multi-nucleated CMs was heterogenous. When separated from the neonatal rat CMs *in vitro*, adult mice CMs dedifferentiated but did not redifferentiate, indicating the importance of physical contact. It was suspected that calcium signalling played a role in this process, as it was suggested that DPR was being stimulated via gap junctions signalling from neighbouring CMs. This may be due to interference with regular calcium signalling within the CM. This study is limited, as it is a cross-species cellular study of mice and rat CMs and does not replicate the *in vivo* scenario. However, it does give valuable insight into the role of intercellular calcium signalling between heterogenous CM populations to induce DPR.

**The Extracellular Matrix (ECM) and Cardiac Regeneration**

The cardiac ECM composition undergoes considerable changes throughout *in utero* development, when CMs are most proliferative, to postnatal maturation, when CMs exhibit limited proliferative capacity^54^ ^10, 19, 54^. Furthermore, the cardiac ECM undergoes distinct changes post-MI, with inflammation, proteolysis and fibrotic scar formation significantly altering infarct and BZECM expression^55, 56^. An early study identified the ECM protein periostin as an inducer of proliferation and CM repair^57^. Periostin is a non-structural matrix protein that shows increased expression in the embryonic^58^ and post-MI heart^59^. Administration of periostin into healthy rat myocardium was shown to induce complete mitosis of adult CMs, acting through integrin receptor and phosphphatidylinositol-3-OH-kinase (PI3K) signalling pathways^60, 61^. Evidence of its regenerative properties were demonstrated when post-MI administration of periostin resulted in improved cardiac function^57^, whilst others showed genetic KO of periostin in mice inhibited post-MI regeneration^62^. Another ECM protein that has been linked to cardiac regeneration is agrin; a large heparan sulfate proteoglycan^63^. Agrin is expressed at high levels within the myocardium P1 and declines rapidly from P1 to P7, correlating with the decline in CM proliferative capacity. In an MI injury model administration of agrin into infarcted mouse hearts not only increased CM proliferation, but also improved heart function, a tenet of cardiac regeneration. This further supports the therapeutic application of ECM proteins to augment CM proliferation in an injury setting (Figure 3).

1. Tan Cheryl Mei J and Lewandowski Adam J. The Transitional Heart: From Early Embryonic and Fetal Development to Neonatal Life. *Fetal Diagnosis and Therapy* 2019; 47: 373-386. DOI: 10.1159/000501906.

2. Eisenberg LM, Kubalak SW and Eisenberg CA. Stem cells and the formation of the myocardium in the vertebrate embryo. *The Anatomical Record Part A: Discoveries in Molecular, Cellular, and Evolutionary Biology: An Official Publication of the American Association of Anatomists* 2004; 276: 2-12.

3. Abu-Issa R and Kirby ML. Heart Field: From Mesoderm to Heart Tube. *Annual Review of Cell and Developmental Biology* 2007; 23: 45-68. DOI: 10.1146/annurev.cellbio.23.090506.123331.

4. Mathew P and Bordoni B. Embryology, heart. *StatPearls [Internet]*. StatPearls Publishing, 2023.

5. Arráez-Aybar LA, Turrero-Nogués A and Marantos-Gamarra DG. Embryonic Cardiac Morphometry in Carnegie Stages 15–23, from the Complutense University of Madrid Institute of Embryology Human Embryo Collection. *Cells Tissues Organs* 2007; 187: 211-220. DOI: 10.1159/000112212.

6. Li-Villarreal N, Rasmussen TL, Christiansen AE, et al. Three-dimensional microCT imaging of mouse heart development from early post-implantation to late fetal stages. *Mammalian Genome* 2023; 34: 156-165. DOI: 10.1007/s00335-022-09976-7.

7. Krishnan A, Samtani R, Dhanantwari P, et al. A detailed comparison of mouse and human cardiac development. *Pediatr Res* 2014; 76: 500-507. 20140828. DOI: 10.1038/pr.2014.128.

8. Steffens S, Nahrendorf M and Madonna R. Immune cells in cardiac homeostasis and disease: emerging insights from novel technologies. *Eur Heart J* 2022; 43: 1533-1541. DOI: 10.1093/eurheartj/ehab842.

9. Radu-Ioniţă F, Bontaş E, Goleanu V, et al. Heart Embryology: Overview. In: Dumitrescu SI, Ţintoiu IC and Underwood MJ (eds) *Right Heart Pathology: From Mechanism to Management*. Cham: Springer International Publishing, 2018, pp.3-24.

10. Li F, Wang X, Capasso JM, et al. Rapid transition of cardiac myocytes from hyperplasia to hypertrophy during postnatal development. *J Mol Cell Cardiol* 1996; 28: 1737-1746. DOI: 10.1006/jmcc.1996.0163.

11. Porrello ER, Mahmoud AI, Simpson E, et al. Transient regenerative potential of the neonatal mouse heart. *Science* 2011; 331: 1078-1080. DOI: 10.1126/science.1200708.

12. Payan SM, Hubert F and Rochais F. Cardiomyocyte proliferation, a target for cardiac regeneration. *Biochimica et Biophysica Acta (BBA) - Molecular Cell Research* 2020; 1867: 118461. DOI: <https://doi.org/10.1016/j.bbamcr.2019.03.008>.

13. Saga Y, Miyagawa-Tomita S, Takagi A, et al. MesP1 is expressed in the heart precursor cells and required for the formation of a single heart tube. *Development* 1999; 126: 3437-3447.

14. Saga Y, Kitajima S and Miyagawa-Tomita S. Mesp1 expression is the earliest sign of cardiovascular development. *Trends in cardiovascular medicine* 2000; 10: 345-352.

15. Costello I, Pimeisl I-M, Dräger S, et al. The T-box transcription factor Eomesodermin acts upstream of Mesp1 to specify cardiac mesoderm during mouse gastrulation. *Nature cell biology* 2011; 13: 1084-1091.

16. Thornburg K, Jonker S, O’Tierney P, et al. Regulation of the cardiomyocyte population in the developing heart. *Progress in Biophysics and Molecular Biology* 2011; 106: 289-299. DOI: <https://doi.org/10.1016/j.pbiomolbio.2010.11.010>.

17. Burrell JH, Boyn AM, Kumarasamy V, et al. Growth and maturation of cardiac myocytes in fetal sheep in the second half of gestation. *The Anatomical Record Part A: Discoveries in Molecular, Cellular, and Evolutionary Biology* 2003; 274A: 952-961. DOI: <https://doi.org/10.1002/ar.a.10110>.

18. Jonker SS, Zhang L, Louey S, et al. Myocyte enlargement, differentiation, and proliferation kinetics in the fetal sheep heart. *Journal of Applied Physiology* 2007; 102: 1130-1142. DOI: 10.1152/japplphysiol.00937.2006.

19. Clubb Jr F and Bishop S. Formation of binucleated myocardial cells in the neonatal rat. An index for growth hypertrophy. *Laboratory investigation; a journal of technical methods and pathology* 1984; 50: 571-577.

20. MacGrogan D, Münch J and de la Pompa JL. Notch and interacting signalling pathways in cardiac development, disease, and regeneration. *Nature Reviews Cardiology* 2018; 15: 685-704. DOI: 10.1038/s41569-018-0100-2.

21. Klaus A, Müller M, Schulz H, et al. Wnt/β-catenin and Bmp signals control distinct sets of transcription factors in cardiac progenitor cells. *Proceedings of the National Academy of Sciences* 2012; 109: 10921-10926.

22. Pahnke A, Conant G, Huyer LD, et al. The role of Wnt regulation in heart development, cardiac repair and disease: A tissue engineering perspective. *Biochem Biophys Res Commun* 2016; 473: 698-703. 20151126. DOI: 10.1016/j.bbrc.2015.11.060.

23. Quaife-Ryan GA, Mills RJ, Lavers G, et al. β-Catenin drives distinct transcriptional networks in proliferative and nonproliferative cardiomyocytes. *Development* 2020; 147: dev193417. DOI: 10.1242/dev.193417.

24. Parikh A, Wu J, Blanton RM, et al. Signaling Pathways and Gene Regulatory Networks in Cardiomyocyte Differentiation. *Tissue Engineering Part B: Reviews* 2015; 21: 377-392. DOI: 10.1089/ten.teb.2014.0662.

25. Fan Y, Ho BX, Pang JKS, et al. Wnt/β-catenin-mediated signaling re-activates proliferation of matured cardiomyocytes. *Stem Cell Res Ther* 2018; 9: 338. 20181207. DOI: 10.1186/s13287-018-1086-8.

26. Prados B, Gómez-Apiñániz P, Papoutsi T, et al. Myocardial Bmp2 gain causes ectopic EMT and promotes cardiomyocyte proliferation and immaturity. *Cell Death & Disease* 2018; 9: 399. DOI: 10.1038/s41419-018-0442-z.

27. Fu W-b, Wang WE and Zeng C-y. Wnt signaling pathways in myocardial infarction and the therapeutic effects of Wnt pathway inhibitors. *Acta Pharmacologica Sinica* 2019; 40: 9-12. DOI: 10.1038/s41401-018-0060-4.

28. Di Baldassarre A, Cimetta E, Bollini S, et al. Human-Induced Pluripotent Stem Cell Technology and Cardiomyocyte Generation: Progress and Clinical Applications. *Cells* 2018; 7 20180525. DOI: 10.3390/cells7060048.

29. Bekhite MM and Schulze PC. Human Induced Pluripotent Stem Cell as a Disease Modeling and Drug Development Platform—A Cardiac Perspective. *Cells* 2021; 10: 3483.

30. Wysoczynski M and Bolli R. A realistic appraisal of the use of embryonic stem cell-based therapies for cardiac repair. *European Heart Journal* 2020; 41: 2397-2404. DOI: 10.1093/eurheartj/ehz787.

31. Li J, Han S, Yu F, et al. Mapping the landscape of PSC-CM research through bibliometric analysis. *Frontiers in Cardiovascular Medicine* 2024; 11. Original Research. DOI: 10.3389/fcvm.2024.1435874.

32. Anderson DJ, Kaplan DI, Bell KM, et al. NKX2-5 regulates human cardiomyogenesis via a HEY2 dependent transcriptional network. *Nature Communications* 2018; 9: 1373. DOI: 10.1038/s41467-018-03714-x.

33. Burgon PG, Weldrick JJ, Talab OMSA, et al. Regulatory Mechanisms That Guide the Fetal to Postnatal Transition of Cardiomyocytes. *Cells* 2023; 12: 2324.

34. Dimasi CG, Darby JRT and Morrison JL. A change of heart: understanding the mechanisms regulating cardiac proliferation and metabolism before and after birth. *The Journal of Physiology* 2023; 601: 1319-1341. DOI: <https://doi.org/10.1113/JP284137>.

35. Ventura-Clapier R, Garnier A, Veksler V, et al. Bioenergetics of the failing heart. *Biochimica et Biophysica Acta (BBA) - Molecular Cell Research* 2011; 1813: 1360-1372. DOI: <https://doi.org/10.1016/j.bbamcr.2010.09.006>.

36. Lopaschuk GD and Jaswal JS. Energy Metabolic Phenotype of the Cardiomyocyte During Development, Differentiation, and Postnatal Maturation. *Journal of Cardiovascular Pharmacology* 2010; 56: 130-140. DOI: 10.1097/FJC.0b013e3181e74a14.

37. Piquereau J and Ventura-Clapier R. Maturation of Cardiac Energy Metabolism During Perinatal Development. *Front Physiol* 2018; 9: 959. 20180719. DOI: 10.3389/fphys.2018.00959.

38. Breckenridge RA, Piotrowska I, Ng K-E, et al. Hypoxic Regulation of Hand1 Controls the Fetal-Neonatal Switch in Cardiac Metabolism. *PLOS Biology* 2013; 11: e1001666. DOI: 10.1371/journal.pbio.1001666.

39. Windmueller R, Leach JP, Babu A, et al. Direct comparison of mononucleated and binucleated cardiomyocytes reveals molecular mechanisms underlying distinct proliferative competencies. *Cell reports* 2020; 30: 3105-3116. e3104.

40. Bae J, Salamon RJ, Brandt EB, et al. Malonate Promotes Adult Cardiomyocyte Proliferation and Heart Regeneration. *Circulation* 2021; 143: 1973-1986. 20210305. DOI: 10.1161/CIRCULATIONAHA.120.049952.

41. Li X, Wu F, Gunther S, et al. Inhibition of fatty acid oxidation enables heart regeneration in adult mice. *Nature* 2023; 622: 619-626. 20230927. DOI: 10.1038/s41586-023-06585-5.

42. Cheng YY, Gregorich Z, Prajnamitra RP, et al. Metabolic Changes Associated With Cardiomyocyte Dedifferentiation Enable Adult Mammalian Cardiac Regeneration. *Circulation* 2022; 146: 1950-1967. 20221124. DOI: 10.1161/CIRCULATIONAHA.122.061960.

43. Senyo SE, Steinhauser ML, Pizzimenti CL, et al. Mammalian heart renewal by pre-existing cardiomyocytes. *Nature* 2013; 493: 433-436. 20121205. DOI: 10.1038/nature11682.

44. Ferreira-Martins J, Ogórek B, Cappetta D, et al. Cardiomyogenesis in the developing heart is regulated by c-kit-positive cardiac stem cells. *Circ Res* 2012; 110: 701-715. 20120124. DOI: 10.1161/circresaha.111.259507.

45. D'Amario D, Leone AM, Iaconelli A, et al. Growth properties of cardiac stem cells are a novel biomarker of patients' outcome after coronary bypass surgery. *Circulation* 2014; 129: 157-172. 20131118. DOI: 10.1161/circulationaha.113.006591.

46. Mollova M, Bersell K, Walsh S, et al. Cardiomyocyte proliferation contributes to heart growth in young humans. *Proc Natl Acad Sci U S A* 2013; 110: 1446-1451. 20130109. DOI: 10.1073/pnas.1214608110.

47. Kirillova A, Han L, Liu H, et al. Polyploid cardiomyocytes: implications for heart regeneration. *Development* 2021; 148: dev199401. DOI: 10.1242/dev.199401.

48. Bersell K, Arab S, Haring B, et al. Neuregulin1/ErbB4 Signaling Induces Cardiomyocyte Proliferation and Repair of Heart Injury. *Cell* 2009; 138: 257-270. DOI: 10.1016/j.cell.2009.04.060.

49. Soonpaa MH, Kim KK, Pajak L, et al. Cardiomyocyte DNA synthesis and binucleation during murine development. *Am J Physiol* 1996; 271: H2183-2189. DOI: 10.1152/ajpheart.1996.271.5.H2183.

50. Bergmann O, Bhardwaj RD, Bernard S, et al. Evidence for cardiomyocyte renewal in humans. *Science* 2009; 324: 98-102. DOI: 10.1126/science.1164680.

51. Bergmann O, Zdunek S, Felker A, et al. Dynamics of Cell Generation and Turnover in the Human Heart. *Cell* 2015; 161: 1566-1575. 20150611. DOI: 10.1016/j.cell.2015.05.026.

52. Yester JW, Liu H, Gyngard F, et al. Use of stable isotope-tagged thymidine and multi-isotope imaging mass spectrometry (MIMS) for quantification of human cardiomyocyte division. *Nat Protoc* 2021; 16: 1995-2022. 20210224. DOI: 10.1038/s41596-020-00477-y.

53. Wang WE, Li L, Xia X, et al. Dedifferentiation, Proliferation, and Redifferentiation of Adult Mammalian Cardiomyocytes After Ischemic Injury. *Circulation* 2017; 136: 834-848. 20170622. DOI: 10.1161/circulationaha.116.024307.

54. Lockhart M, Wirrig E, Phelps A, et al. Extracellular matrix and heart development. *Birth Defects Research Part A: Clinical and Molecular Teratology* 2011; 91: 535-550.

55. Dobaczewski M, Gonzalez-Quesada C and Frangogiannis NG. The extracellular matrix as a modulator of the inflammatory and reparative response following myocardial infarction. *Journal of molecular and cellular cardiology* 2010; 48: 504-511.

56. Frangogiannis NG. Pathophysiology of myocardial infarction. *Comprehensive Physiology* 2011; 5: 1841-1875.

57. Kuhn B, del Monte F, Hajjar RJ, et al. Periostin induces proliferation of differentiated cardiomyocytes and promotes cardiac repair. *Nat Med* 2007; 13: 962-969. 20070715. DOI: 10.1038/nm1619.

58. Kruzynska-Frejtag A, Machnicki M, Rogers R, et al. Periostin (an osteoblast-specific factor) is expressed within the embryonic mouse heart during valve formation. *Mech Dev* 2001; 103: 183-188. DOI: 10.1016/s0925-4773(01)00356-2.

59. Zhao S, Wu H, Xia W, et al. Periostin expression is upregulated and associated with myocardial fibrosis in human failing hearts. *Journal of Cardiology* 2014; 63: 373-378. DOI: <https://doi.org/10.1016/j.jjcc.2013.09.013>.

60. Gilbert CJ, Longenecker JZ and Accornero F. ERK1/2: an integrator of signals that alters cardiac homeostasis and growth. *Biology* 2021; 10: 346.

61. Walkowski B, Kleibert M, Majka M, et al. Insight into the role of the PI3K/Akt pathway in ischemic injury and post-infarct left ventricular remodeling in normal and diabetic heart. *Cells* 2022; 11: 1553.

62. Chen Z, Xie J, Hao H, et al. Ablation of periostin inhibits post-infarction myocardial regeneration in neonatal mice mediated by the phosphatidylinositol 3 kinase/glycogen synthase kinase 3β/cyclin D1 signalling pathway. *Cardiovascular Research* 2017; 113: 620-632. DOI: 10.1093/cvr/cvx001.

63. Bassat E, Mutlak YE, Genzelinakh A, et al. The extracellular matrix protein agrin promotes heart regeneration in mice. *Nature* 2017; 547: 179-184. 20170605. DOI: 10.1038/nature22978.
